# Mental Health Symptoms and Work-Related Stressors in Hospital Midwives and NICU Nurses: A Mixed Methods Study

**DOI:** 10.3389/fpsyt.2018.00364

**Published:** 2018-08-20

**Authors:** Céline Favrod, Lauranne Jan du Chêne, Chantal Martin Soelch, Susan Garthus-Niegel, Jean-Francois Tolsa, Francois Legault, Virginie Briet, Antje Horsch

**Affiliations:** ^1^Department Woman-Mother-Child, Lausanne University Hospital, Lausanne, Switzerland; ^2^Department of Psychology, University of Fribourg, Fribourg, Switzerland; ^3^Department of Psychotherapy and Psychosomatic Medicine, Faculty of Medicine, Technische Universität Dresden, Dresden, Germany; ^4^Department of Obstetrics and Gynecology, Geneva University Hospitals (HUG), Geneva, Switzerland; ^5^Institute of Higher Education and Research in Healthcare (IUFRS), University of Lausanne, Lausanne, Switzerland

**Keywords:** anxiety, depression, secondary traumatic stress, burnout, professional, stressor, midwives, nurses

## Abstract

Hospital midwives and neonatal intensive care (NICU) nurses frequently encounter work-related stressors and are therefore vulnerable to developing mental health problems, such as secondary traumatic stress, burnout, anxiety, and depression. However, so far, the exact nature of these work-related stressors (traumatic vs. non-traumatic stressors) has not been investigated. This concurrent triangulation mixed methods cross-sectional study aimed to compare mental health symptoms in hospital midwives and NICU nurses, and to identify and compare work-related traumatic and non-traumatic stressors for both professional groups. 122 midwives and 91 NICU nurses of two Swiss university hospitals completed quantitative measures (Secondary Traumatic Stress Scale, STSS; Hospital Anxiety and Depression Scale, HADS; Maslach Burnout Inventory, MBI) and one qualitative question in an online survey. When controlling for socio-demographic variables, NICU nurses had a higher STSS total score and higher STSS subscales scores and less HADS anxiety subscale scores than hospital midwives. Work-related stressors were classified into five themes: “Working environment,” “Nursing/midwifery care,” “Dealing with death and dying,” “Case management” and “Others.” Forty-six (46.3%) percent of these were classified as traumatic work-related stressors. NICU nurses reported more traumatic stressors in their working environment but no other differences between professional groups regarding the total number of work-related traumatic vs. non-traumatic stressors were found. Measures, such as teaching strategies to amend the subjective appraisal of the traumatic stressors or providing time to recover in-between frequently occurring work-related traumatic stressors might not only improve the mental health of professionals but also decrease sick leave and improve the quality of patient care.

## Introduction

Work-related psychosocial stress is one of the most concerning issues of occupational health in industrialized countries (1). Stress, experienced by approximately 45% of working Europeans, is seen as the second most important threat posed by the working environment (after musculoskeletal problems), and costs approximately 25 billion euros per year (2). Fourteen percent of persons suffering from work-related health difficulties report stress, depression or anxiety as their severest health problem (2). As a result, reducing the burden of work-related mental health problems and psychiatric sickness absence is a key priority for the World Health Organization (3). Among hospital workers, patient-care professionals are more vulnerable than other professionals to develop mental health difficulties (4). Their mental health problems are linked to high quantitative, emotional, sensorial and cognitive demands at work, a high rhythm of work, and a demand for hiding emotions (4).

The present study focuses on staff working with the perinatal population in a hospital environment: midwives working in a maternity department and nurses working in a neonatal intensive care unit (NICU). For midwives, insufficient time to do what needs to be done and inability to change work-based decisions (made by midwifery colleagues or doctors) were identified as the two major types of stress (5). In one study, the majority of midwives considered their job as stressful and thought that lack of work resources and poor organization at work caused the most stress (6). Furthermore, midwives working in hospitals felt less supported than independent midwives (7). They struggled between being with the women and meeting service needs (8). Midwives also reported insufficient support from co-workers (9). Approximately two third of midwives tend to suffer from burnout (10, 11). Midwives in developing countries are frequently faced with maternal death. Within a population of Ugandan midwives who experienced maternal death, 20% reported high anxiety (12).

Nursing in neonatal intensive care is very demanding and linked with moral distress (13). Intensive care staff is regarded as particularly vulnerable compared to other healthcare professionals, as they encounter additional stressors compared to those working in other areas, regularly face ethical dilemmas concerning patient care management, and the threat of committing errors that may have serious consequences (14, 15). Acute stressors such as dealing with dying and with death, dealing with young patients and responding to critical situations are frequent (16) and staff often have to move from one traumatic event to another, leaving little time for recovery (17, 18). Even though a previous study indicated that NICU nurses caring for the dying newborn and the newborn's parents learn to deal with those difficult situations and seek strategies to limit their suffering (16), there is very limited evidence and research investigating the mental health of NICU nurses is lacking.

We propose that work-related stressors, which hospital midwives and NICU nurses frequently encounter, can be classified into *traumatic* stressors, defined by direct or indirect exposure to death, threatened death, actual or threatened serious injury, actual or threatened sexual violence (18–20) and *non-traumatic* stressors, such as the lack of regular shifts, a heavy workload, and limited resources.

Being exposed to work-related traumatic stressors may render midwives and NICU nurses vulnerable to developing secondary traumatic stress disorder (STSD) symptoms following “repeated or extreme indirect exposure to aversive details in the course of professional duties” (21). Secondary traumatic stress disorder is diagnosed when the professional is exposed to the first hand trauma experiences of a patient and is characterized by re-experiencing, avoidance, and hyperarousal symptoms (22). The symptoms of STSD are the same as those of posttraumatic stress disorder (PTSD) in the fourth edition of the Diagnostic and Statistical Manual of Psychiatric Disorders (DSM-IV-TR; 23). However, unlike PTSD, STSD is due to indirect exposure in a professional context. It was not included in DSM-IV-TR as a formal psychiatric diagnosis. In DSM-5 (21), the new traumatic stressor criterion A4 highlights professional responsibilities as potential traumatic stressors that could trigger PTSD.

Midwives and NICU nurses are both vulnerable to developing STSD because they both encounter traumatic work-related stressors. Midwives regularly have to manage traumatic births and other traumatic perinatal events (24), which can cause secondary traumatic stress (25) and post-traumatic stress disorder (PTSD) (26). A postal survey in the UK found that over 95% of midwives had been directly or indirectly exposed to a traumatic event at work (11). Midwives' principal fears are related to death (of the baby or the mother), medical emergency and being the cause of a negative birth experience (27). In addition, it has been argued that due to their close relationship with the women, they are vulnerable to compassion fatigue, which is also linked with STSD (25). What is more, evidence shows that high empathy in midwives is associated with a higher risk of PTSD Sheen et al. (11). Whilst research focusing on STSD specifically in NICU nurses is lacking, some studies in ICU nurses have reported STSD and PTSD symptoms (17, 18, 28).

Therefore, the aims of our study were (a) to assess the prevalence rates of mental health symptoms in NICU nurses and midwives working in a hospital environment, (b) to compare those groups regarding their mental health symptoms, and (c) to identify and compare work-related traumatic and non-traumatic stressors for both professional groups.

## Methods

### Participant consent and recruitment

The study took place in two university hospitals in the French-speaking part of Switzerland using the same recruitment procedure: midwives and NICU nurses were informed about the study during staff meetings and by flyers. All eligible participants were systematically paid an extra hour of work to encourage their participation. Staff accessing the anonymous online survey [LimeSurvey (version 2.0) (29)] found a detailed information sheet before giving informed consent. The survey consisted of five questionnaires and took approximately 30 min to complete. All eligible participants received one reminder e-mail before the survey closed. Ethical approval was obtained from the ethics committee of the Canton de Vaud, Switzerland (study nr: 237/2013).

### Procedure

Of the 209 eligible midwives, 125 participated (59.8% response rate). One-hundred and 22 (58.4%) completed a sufficient number of items to allow the replacement of missing data (see data analysis for more details). All but one midwife (122, 99.2%) responded to the qualitative question. Of the 170 eligible NICU nurses, 91 participated (53.5% response rate). Of those, 84 (49%) completed a sufficient number of items of the questionnaires to allow replacement of missing data. Forty-nine (54%) NICU nurses responded to the qualitative question.

### Design

A concurrent triangulation mixed methods cross-sectional design including quantitative and one qualitative question in an online survey was employed (30). Qualitative and quantitative data were collected concurrently. They were analyzed separately and then combined (30).

### Instruments

#### Quantitative approach

Secondary traumatic stress was measured using the Secondary Traumatic Stress Scale (STSS) (22), a self-report questionnaire specifically designed for professional caregivers, with the instruction and the stems of eight stressor-specific items referring explicitly to “client exposure” as traumatic stressor. Based on the definition of PTSD in DSM-IV-TR, the STSS consists of three subscales: intrusion (5 items), avoidance (7 items), and arousal (neurovegetative activation; 5 items) in a total of 17 items with a Likert scale of five points (1 “*never*” to 5 “*very often*”). The time period of measured symptoms is the last seven days. The total score is calculated by adding the total of the three subscales, with a high score indicating a higher level of symptoms (31, 32). A score below 28 corresponds to “little or no secondary traumatic stress,” a score between 28 and 37 means “mild secondary traumatic stress,” between 38 and 43 “moderate secondary traumatic stress,” between 44 and 48 “high secondary traumatic stress,” and beyond 49 “severe secondary traumatic stress;” the score of 38 is used as critical threshold indicating secondary traumatic stress disorder (19). This questionnaire has good psychometric properties (33). The scale was recently validated in a sample of Swiss midwives (Jacobs et al., under review). In this study, using the recently validated version, the Cronbach's alpha was α = 0.903.

Anxiety and depression symptoms were measured using the French version of the Hospital Anxiety and Depression Scale (HADS) (34–37), which is a 14-item self-report questionnaire measuring state anxiety (7 items) and depression (7 items) with good psychometric properties. The time period of measured symptoms is the last seven days. Each item is calculated from 0 to 3, with higher scores indicating higher anxiety and/or depression. For both, the anxiety and depression subscale, a score between 8 and 10 indicates a possible clinical disorder, and a score between 11 and 21 indicates a probable clinical disorder (38, 39). The HADS can also be used as scale of anxiety or depression symptom severity, ranging from normal (0–7), low (8–10), moderate (11–14), and severe (15–21), with a critical threshold at 11 for both subscales. In this study the Cronbach's alpha was α = 0.814.

Burnout was measured using the Maslach Burnout Inventory (40) which is a self-report questionnaire with 22 items rated on a Likert scale from 0 “*never*” to 6 “*always*”. The items are organized into three subscales; “emotional exhaustion” (i.e., feeling emotionally overextended and exhausted by one's work; 9 items), which is the core dimension (41, 42), “depersonalization” (i.e., impersonal response toward recipients of one's service or care treatment; 5 items), and “personal achievement” (i.e., feeling competent and successful in one's work; 8 items). The timeframe of experienced burnout measured is not indicated (general frequency, from never to every day). Scores are calculated separately for each subscale (9). A score >30 on the first subscale is considered as a severe burnout, severe depersonalization is considered with a score >12, and severe deficiency in personal achievement with a score < 33 and critical thresholds are the moderate levels for each subscales (43). The validated French version of this questionnaire was used (44). In the present study the Cronbach's alpha were, 0.872 for the emotional exhaustion subscale, 0.643 for the depersonalization subscale and 0.678 for the personal achievement subscale.

In addition, participants responded to questions regarding their gender, age, country of origin, years of work experience (less or more than 10 years), form of employment (full-time or part-time work), and marital status.

#### Qualitative approach

Participants were asked to list examples of traumatic situations they had experienced at either in the NICU or on the labor ward in the past year. (“Please describe briefly work-related stressors you have encountered at work in the past year”).

### Data analysis

#### Quantitative approach

Quantitative questionnaire data were analyzed with IBM SPSS statistics 22 software (Statistical Package for Social Sciences). Missing data analysis was performed (45) when less than 50% of the items per subscale were missing (46). For each subscale the null hypothesis of random missing data distribution was tested using Little's MCAR test (45). If the null hypothesis was not rejected, we proceeded to imputation calculation using the Expectation-maximization of missing value process. However, if ≥50% of the items per subscale were missing, missing data were not replaced. Descriptive statistics were calculated for each scale. The percentage of missing data not replaced (when 50% or more of the item of a subscale were missing) varied between 7.7 and 9.9% for NICU nurses and between 1.6 and 2.5% for midwives. The group differences analyses were repeated without replacement of the missing data and no main differences were found (data not shown).

Given that not all NICU nurses in the sample responded to the qualitative question concerning work-related stressors and in order to detect potential biases, responders and non-responders were compared regarding socio-demographic variables and mental health symptoms. Chi-squared analyses were performed for categorical variables across professional groups (gender, age, country of origin, years of work experience, work participation, relationship status), *t*-tests were performed for normally distributed continuous variables (MBI emotional exhaustion and personal achievement subscales). Finally, Mann-Whitney rank tests were performed for non-normally distributed continuous variables (HADS anxiety and depression subscales, STSS total, STSS intrusion, avoidance and arousal subscales as well as MBI depersonalization subscale).

Normality was tested for continuous variables using Kolmogorov-Smirnoff criteria. To compare professional groups, independent samples *t*-tests were performed with continuous variables that were normally distributed (MBI subscales “emotional exhaustion” and “personal achievement”) and non-parametric independent-samples Mann-Witney *U*-tests were performed for variables not normally distributed (STSS, STSS subscales, HADS-anxiety, HADS-depression, and MBI subscale “depersonalization”). Chi-squared tests were run to compare categorical variables between groups. All reported *p*-values are 2-tailed and the effect size for the Mann-Whitney *U*-test was calculated as follows: U/(n_NICUnurses_^*^ n_midwifes_) (47).

When sample characteristics differed significantly between groups, stepwise regression analyses were run to control the influence of these variables on mental health symptoms that differed between professional groups.

#### Qualitative approach

For the qualitative question, a content analysis based on the manifest content of the written examples of work-related stressors provided by both midwives and NICU nurses was performed by two independent raters (CF, LJdC) (48). Coding categories were created and their frequency was counted, with the entire interview used as a coding unit (49). Firstly, the raters read each answer in order to obtain a general impression. Secondly, each given example was condensed, then organized into sub-categories (based on similarities and differences of condensed given examples). Once the raters agreed on the sub-categories, they independently sorted them into categories and then looked for agreement on these. Following this, a second classification was performed sorting each given example into the following two categories: “traumatic work-related stressor” if the given example met the diagnostic DSM 5 criterion A for PTSD (21) and “non-traumatic work-related stressor” if it did not respond to this criterion. Therefore, categories could contain traumatic as well as non-traumatic work-related stressors. Descriptive statistics analyses were run for both classifications as well as comparisons between midwives and NICU nurses. Chi squared analyses were run to compare group distributions within each categories as well as distributions between “Traumatic work-related stressor” and “non-traumatic work-related stressor.”

## Results

### Sample

For both professional groups, the majority of participants were women. About half of the midwives and the majority of NICU nurses were aged between 26 and 40 years. Most midwives worked part-time, whereas most NICU nurses worked full-time. Approximately 40% of midwives and 55% of NICU nurses had 10 years or less experience, whereas ~60% of midwives and 43% of NICU nurses had more than 10 years of experience. More socio-demographic details are presented in Table 1. Group comparisons revealed no differences regarding gender or country of origin, but significant differences for age (*p* < 0.001), years of work experience (*p* = 0.024), work participation (*p* < 0.001), and relationship status (*p* = 0.003).

**Table 1:** Demographic sample characteristics and group comparisons.

	**NICU nurses (*****N*** = **91)**		**Midwives (*****N*** = **122)**		Group differences analyses^*^		
	***n***	***%***	***n***	***%***	**$\chi_{\left(df\right)}^{2}$**	***p***	**Effect size φ**
Gender					$\chi_{\left(1\right)}^{2}$ = 1.32	0.250	
Men	5	5.5	3	2.5			
Women	84	92.3	116	95.1			
Missing values	2	2.2	3	2.5			
Age					$\chi_{\left(3\right)}^{2}$ = 16.26	<**0.001**	0.280
18 to 25 years old	8	8.8	3	2.5			
26 to 30 years old	24	26.4	21	17.2			
31 to 40 years old	40	44	41	33.6			
> 40 years old	18	19.8	53	43.4			
Missing values	1	1.1	4	3.3			
Country of origin					$\chi_{\left(3\right)}^{2}$ = 5.61	0.132	
Switzerland	36	39.6	59	48.4			
Other EU countries	43	47.2	57	46.7			
Non-EU countries	11	12.1	6	4.9			
Missing values	1	1.1	0	0			
Years of work experience					$\chi_{\left(1\right)}^{2}$ = 5.13	<**0.050**	0.157
≤ 10 years	50	54.9	48	39.3			
> 10 years	39	42.9	71	58.2			
Missing values	2	2.2	3	2.5			
Work participation					$\chi_{\left(1\right)}^{2}$ = 31.40	<**0.001**	0.386
Part-time	34	37.4	92	75.4			
Full-time	56	61.5	29	23.8			
Missing values	1	1.1	1	0.8			
Relationship status					$\chi_{\left(3\right)}^{2}$ = 13.86	<**0.010**	0.255
Single	42	46.2	38	31.1			
Partnered	49	53.8	84	68.9			

* *Group differences are examined estimating chi square (χ^2^) differences. Bold: p < 0.05*.

### Group comparisons regarding mental health symptoms

**Figure 1:**
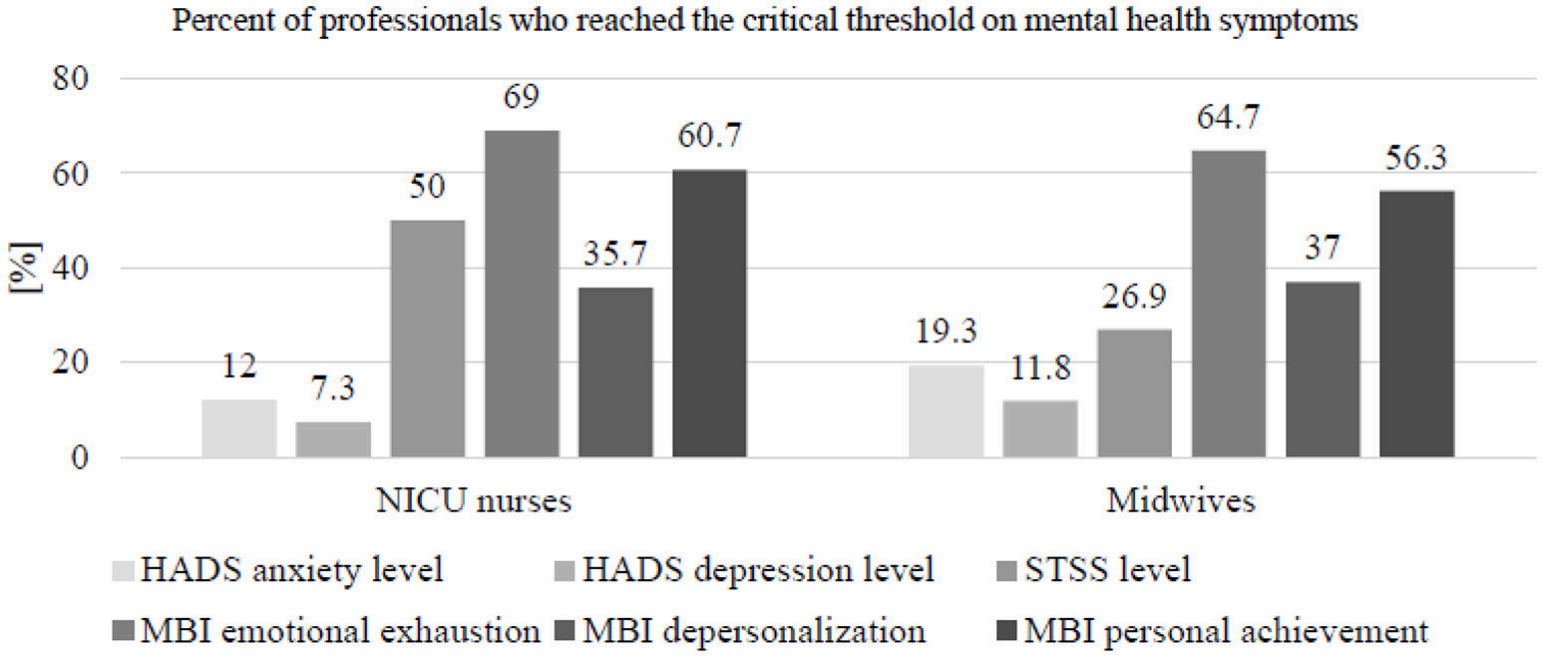
Importance of mental health symptoms by professional group. Severity levels and critical thresholds are defined by authors of each test as follows: HADS anxiety and HADS depression: moderate or severe symptoms. STSS: moderate, high or severe symptoms. MBI: moderate or high symptoms.

Table 2 presents mean scores and standard deviations of mental health symptoms in midwives and NICU nurses. Group comparisons revealed that NICU nurses had a higher STSS total score than midwives (*p* < 0.001), and also higher scores for all three STSS subscales: intrusion (*p* = 0.002), avoidance (*p* < 0.001), and arousal (*p* = 0.002). Similarly, NICU nurses were more likely to reach higher STSS severity (high and severe) levels (*p* < 0.001). No significant group differences concerning MBI subscales scores and severity levels were found, but NICU nurses were more likely to have severe global burnout than midwives (*p* = 0.016). On the other hand, midwives had a higher HADS anxiety score (*p* = 0.004), were more likely to reach a high HADS anxiety severity level (*p* = 0.012), and had a higher HADS depression score (*p* = 0.041). However, no group difference was found regarding the HADS depression severity level (*p* = 0.098). Figure 1 presents the percentages of each professional group affected by mental health symptoms.

**Table 2:** Mean scores and standard deviations of psychopathological symptoms and group comparisons.

	**NICU nurses (*****n*** **=** 84^*^)			**Midwives (*****n*** **=** 120^*^)			**Group differences analyses**		
	**Mean**	**SD**	**%**	**Mean**	**SD**	**%**	**$\chi_{\left(df\right)}^{2}$;U or t_**(df)**_**	***p***	Effect size^**^
HADS Anxiety total scores	6.3	3.2		7.7	3.8		*U* = 3775.5	** < 0.010**	0.382
HADS Anxiety severity level							$\chi_{\left(3\right)}^{2}$ = 10.974	** < 0.050**	0.233
Normal			74.7			52.9			
Low			13.3			27.7			
Moderate			10.8			14.3			
Severe			1.2			5.0			
HADS Depression total scores	3.9	3.4		5.1	3.8		*U* = 4107.0	** < 0.050**	0.416
HADS Depression severity level							$\chi_{\left(2\right)}^{2}$ = 4.642	0.098	
Normal			84.3			71.4			
Low			8.4			16.8			
Moderate			7.3			11.8			
Severe			0			0			
STSS total scores	38.7	10.9		31.8	9.7		*U* = 3116.0	** < 0.001**	0.319
STSS intrusion total scores	11.1	4.0		9.5	3.7		*U* = 3635.5	** < 0.010**	0.373
STSS avoidance total scores	15.1	4.7		11.7	3.7		*U* = 2810.5	** < 0.001**	0.285
STSS arousal total scores	12.4	4.0		10.6	3.7		*U* = 3706.0	** < 0.010**	0.375
STSS severity level							$\chi_{\left(4\right)}^{2}$ = 20.764	** < 0.001**	0.321
Little or none			17.1			43.7			
Mild			32.9			29.4			
Moderate			22.0			16.8			
High			8.5			4.2			
Severe			19.5			5.9			
MBI severe burnout on all MBI subscales							$\chi_{\left(1\right)}^{2}$ = 5.781	** < 0.050**	0.169
Yes			4.8			0			
No			95.2			100			
MBI emotional exhaustion scores	23.0	9.9		20.7	8.7		*t*_(201)_ = 1.758	0.080	
MBI emotional exhaustion severity level							$\chi_{\left(2\right)}^{2}$ = 5.032	0.081	
Low			31.0			35.3			
Moderate			47.6			54.6			
High			21.4			10.1			
MBI depersonalization scores	4.8	4.1		4.8	3.8		*U* = 4931.0	0.870	
MBI depersonalization severity level							$\chi_{\left(2\right)}^{2}$ = 3.075	0.215	
Low			64.3			63.0			
Moderate			29.8			35.3			
High			6.0			1.7			
MBI personal achievement scores^a^	31.6	5.5		32.9	4.1		*t*_(201)_ = 1.771	0.078	
MBI personal achievement severity level							$\chi_{\left(2\right)}^{2}$ = 1.089	0.580	
High			3.6			6.7			
Moderate			35.7			37.0			
Low			60.7			56.3			

* *Due to missing values on some of the items, n varied between 82 and 84 for NICU nurses, respectively between 119 and 120 for midwives*.

*HADS, Hospital Anxiety and Depression Scale; STSS, Secondary Posttraumatic Stress Scale; MBI, Maslach Burnout Inventory*.

*bold: p < 0.05*.

** *Effect size calculations depend on variable type: **φ** for categorical variables; d for normally distributed continuous variables; **ρ** for not normally distributed ordinal variables*.

a *A low score indicates low personal achievement and is an indicator of burnout*.

Multiple regression analyses were conducted (see Table 3) to see if socio-demographic variables that were significantly different between professional groups (age category, years of experience, work participation, and relationship status) predicted STSS total and subscale scores or the HADS subscale scores. Using the stepwise method, it was found that only the professional group explained a significant amount of the variance of the STSS total score of the STSS avoidance subscale score, of the STSS arousal subscale score and of the HADS anxiety subscale score. Age category and professional group together influenced the STSS intrusion subscale score. Finally, a last regression analysis showed that the HADS depression subscale score was influenced by age category and work participation and that the professional group had no significant influence.

**Table 3:** Multiple regression analyses.

	**STSS total score β**	**STSS avoidance β**	**STSS arousal β**	**STSS intrusion β**	**HADS anxiety β**	**HADS depression β**
Age	0.001	0.118	0.085	−0.189^**^	0.032	0.254^**^
Years of experience	−0.040	0.072	0.017	−0.139	−0.036	0.031^*^
Work participation	0.049	−0.014	0.038	0.039	0.023	0.175^*^
Relationship status	0.039	0.075	0.058	0.057	0.083	0.032^*^
Professional group	−0.335^***^	−387^***^	−0.236^**^	−0.169^*^	0.165^*^	0.134
*R*^2^	0.112	0.150	0.056	0.080	0.027	0.050

* *p < 0.05*,

** *p < 0.01*,

*** *p < 0.001. Bold: p < 0.05*.

In summary, when controlling for socio-demographic variables, NICU nurses had a higher STSS total score and higher STSS subscales scores and less HADS anxiety subscale scores than hospital midwives.

### Responders vs. non-responders to qualitative question

One-hundred-and-eleven midwives gave 516 examples of work-related stressors, one midwife did not answer the question and 10 responded that they had no example to cite (response rate: 99.2%). As only one midwife did not respond, no comparisons between responders and non-responders were run. One hundred-twenty-three examples of work-related stressors were given by 49 (54%) NICU nurses. Comparing NICU nurses who gave at least one example of a work-related stressor with the ones who did not, showed no significant differences regarding socio-demographic variables (gender, age category, country of origin, years of work experience, work participation, or relationship status). Regarding mental health symptoms, there were significant differences: NICU nurses who gave at least one example of a work-related stressor had higher symptoms than those who did not provide an example for STSS total scores (*p* = 0.018) and STSS intrusion subscale (*p* = 0.011). NICU nurses who gave at least one example of a work-related stressor reported higher symptoms on the MBI subscale “emotional exhaustion” than those who did not provide an example (*p* = 0.0.23), as well as higher symptoms on the HADS depression subscale (*p* = 0.049). No other differences regarding mental health outcomes were found (all *p* = ns). Details are provided in Table S1 in Supplementary Data.

### Work-related stressors

The written manifest content analysis of the work-related stressors resulted in five categories: “Working environment,” “Nursing/midwifery care,” “Dealing with death and dying,” “Case management,” and “Others” (Tables 4, 5). The largest category “Nursing/midwifery care” contained 231 situations (36.1%) in total. This category contained situations concerning care procedures and patients' medical situations (pathology, care procedure, medical errors, ethical concerns about care) as well as professional competences, e.g., “*Obstetric situation with difficult outcome, e.g., cesarean emergency with poor recovery of the fetal heart rate.”* The second largest category “Working environment” included 205 situations (32.1%) in total. These situations were related to the organization of work and relations with medical staff (physicians, co-workers, workers from other wards, as well as superiors) or were seen as consequences of a lack of work-organization or poor relationships among staff, e.g., “*Work overload and lack of personnel.”* The category “Dealing with death and dying” comprised 118 situations (18.5%). It contained situations linked with the death of patients, taking care of dying patients, assistance for grieving patients. e.g., “*Quick death of a patient without possibility of help.”* The category “Case management” consisted of 75 situations (11.7%). It included situations related to difficulties in relationships with patients or patients' relatives, and the management of difficult psychosocial situations, e.g., “*Never happy, very demanding and difficult parents, who see us as servants, even after all the effort we made for them.”* Finally, the category “Other” contained 10 situations (1.6%). These were situations that did not occur on the actual ward but stemmed from work experiences in other departments, e.g., “*Resuscitation of a one-year old child who nearly drowned and who died later.”*

**Table 4:** Categorization of work-related stressful situations.

	**Traumatic situations**			**Non-traumatic situations**		
	NICU nurses *n* (%^*^)	Midwives *n* (%^*^)	**Total *n* (%)**	NICU nurses *n* (%^*^)	Midwives *n* (%^*^)	**Total *n* (%)**
Working environment	7 (11.5%)	4 (1.7%)	11 (3.7%)	35 (56.4%)	159 (56.6%)	194 (56.6%)
Nursing/ midwifery care	20 (32.8%)	125 (53.2%)	145 (49.0%)	17 (27.4%)	69 (24.6%)	86 (25.1%)
Dealing with death and dying	28 (45.9%)	90 (38.3%)	118 (39.9%)	0	0	0
Case management	4 (6.6%)	16 (6.8%)	20 (6.7%)	6 (9.7%)	49 (17.4%)	55 (16.0%)
Others	2 (3.3%)	0	2 (0.7%)	4 (6.5%)	4 (1.4%)	8 (2.3%)
Total	61	235	296 (46.3%)	62	281	343 (53.7%)

* *Percent within professional groups*.

**Table 5:** Examples of traumatic and non-traumatic work-related stressors.

	**Traumatic situations**		**Non-traumatic situations**	
	**NICU nurses**	**Midwives**	**NICU nurses**	**Midwives**
Working environment	Resuscitation in the delivery room of a newborn at term (in connection with poor management of childbirth)	A long and significant deceleration of the fetus' heartbeat during the ultrasound without the possibility of calling for help or stopping the current examination because no nearby alarm	Unable to support (help) patients and especially the parents for lack of time	Lack of staff for emergencies
Nursing/midwifery care	Emergency intubation - very difficult	Neonatal resuscitation	Fear of having to take care of a case that is too difficult, not to be in control of the situation, not to observe important signs that should make me worry about the state of health of the patient	Shoulder dystocia
Dealing with death and dying	Death of a term baby due to asphyxia	Maternal death	–	–
Case management	Resuscitation of a child of 6 months, deceased (child shaken by the father)	Death threats made by the husband of a patient giving birth	A parent who becomes aggressive	Having to manage a complex patient living in social and psychological precariousness
Others	Massive digestive hemorrhage when working with adults	–	Clinical teaching (teaching and evaluation at the same time)	Waiting to manage a situation that was announced without being able to act (receiving a telephone call and waiting that the patient arrives)

### Traumatic vs. non-traumatic work-related stressors and group comparisons

Categorizing these 639 work-related stressor examples into traumatic vs. non-traumatic work-related stressors (according to DSM 5) resulted in 296 (46.3%) traumatic work-related stressors, e.g., “*Death of patient. We believe that the experience is stored in a closet but the door opens regularly under excess pressure and all the memories of other deaths reappear.”*, and 343 (53.7%) non-traumatic work-related stressors, such as “*Not being able to accompany patients, and especially the parents, for lack of time.”* “Nursing/midwifery care” contained the highest number of traumatic work-related stressors (*n* = 145), and “Working environment” the highest number of non-traumatic work-related stressors (*n* = 194; see Tables 4, 5 for more details and examples) as well as the lowest number of traumatic stressors (*n* = 11). Group comparisons regarding the total number of traumatic vs. non-traumatic stressors showed no significant difference between professional groups [χ^2^_(1)_ = 0.655; *p* = 0.418]. However, a difference between midwifes and NICU nurses concerning the number of traumatic vs. non-traumatic work-related stressors for one category, the “Working environment,” was found, with NICU nurses reporting more traumatic work-related stressors than midwives [χ^2^_(1)_ = 13.28; *p*<*0*.001] within this category.

## Discussion

This study assessed and compared mental health symptoms in hospital midwives and NICU nurses, and identified and compared work-related traumatic and non-traumatic stressors for both professional groups. Results showed that midwives and NICU nurses respectively reported high levels of secondary traumatic stress, burnout and anxiety symptoms. Interestingly, NICU nurses reported more secondary traumatic stress than midwives but midwives suffered from more anxiety. Midwives' and NICU nurses' work-related stressors were categorized into “Working environment,” “Nursing/midwifery care,” “Dealing with death and dying,” “Case management,” and “Others.” However, there were no differences between professional groups regarding the total number of work-related traumatic vs. non-traumatic stressors, except for “Working environment,” where NICU nurses reported more traumatic situations.

Prevalence rates of STSD symptoms in our study were significantly different between professional groups, with 26.9% of midwives and 50% of NICU nurses reporting symptoms above the critical threshold. This difference remained when controlling for socio-demographic variables. Our prevalence rates of midwives are comparable to other studies in which approximately one-third reported STSD (19, 50, 51). So far, prevalence rates of STSD for NICU nurses have not been reported but studies focusing on emergency nurses found a lower prevalence (e.g., 15%) (17). It appears that NICU nurses are at a higher risk of developing STSD than midwives because they encounter more frequent traumatic work-related stressors (14, 17, 28, 52–54). Staff working in intensive care units (ICUs), like NICU nurses, are regarded as particularly vulnerable compared to other healthcare professionals, as they encounter additional stressors than those working in other areas: regularly face ethical dilemmas concerning patient care management, are regularly confronted to patients dying as well as medical errors that can have serious consequences (14, 15).

In order to examine the nature of the stressors in more detail, we analyzed the type of work-related stressors encountered by both professional groups. We found that 46.3% of all stressors could be classified as traumatic work-related and 53.7% as non-traumatic work-related stressors according to the DSM 5 definition. Interestingly, there were no differences between professional groups regarding the total number of work-related traumatic vs. non-traumatic stressors, except for “Working environment” where NICU nurses reported more traumatic situations. Nevertheless, only few traumatic situations fell within the category “Working environment” (11.5%). A recent publication including midwives showed that the subjective interpretation of the stressors as well as the receipt of support following the stressors had more impact on the mental health than the objective nature of the stressor. There is also some evidence that NICU nurses have little time to recover and to seek support between their frequent encounters of traumatic events (16–18). This might partly explain our results and future research should assess both the subjective interpretation of the stressors as well as the role of social support. However, our results need to be interpreted with caution, as NICU nurses who responded to this question had higher HADS depression symptoms, STSS symptoms, and higher symptoms on the MBI subscale “emotional exhaustion” than non-responders.

Anxiety symptoms were significantly higher in midwives than in NICU nurses (with 19.3 and 12% respectively, scoring above the critical threshold); this difference also remained when controlling for socio-demographic variables. The rate of anxiety symptoms we found for NICU nurses is consistent with previous research on ICU nurses (18, 28, 55). Comparisons regarding anxiety levels in midwives with other studies were not possible due to a lack of published studies. However, one Ugandan study showed that having witnessed maternal death was a predictor of death anxiety in midwives (12). These results can, of course, not easily be transposed to our European context where maternal death is rare with a ratio of five maternal deaths per 100'000 live births in Switzerland against 343 in Uganda (56). Still, the examples of work-related traumatic stressors in our study showed that when a maternal death happened, it also likely affected midwives who had not directly been involved with the case. The death of an adult more than the death of a newborn might trigger anxieties linked to one's own death (12) and might therefore be linked to higher anxiety symptoms in midwives compared to NICU nurses. Indeed, midwives' principal fears are related to death (of the baby or the mother), medical emergency and being the cause of a negative birth experience (27). It is also likely that factors related to their professional role and working environment [such as a low level of perceived control (57)] make midwives more vulnerable to developing anxiety symptoms. The essence of midwives' professional role is to “be with the woman” (7) and hospital midwives working under the dominant biomedical model struggle to maintain this primary professional value while responding to service pressures (7, 8). Indeed, a recent study identified that the fear of being watched and criticized was one of the most prevalent fears in midwives (27).

In addition, midwives also reported a higher total mean score of depressive symptoms than NICU nurses but this difference disappeared when controlling for socio-demographic variables. Indeed, regression analyses showed that the association to the HADS depression score was larger to age and percentage of work more than to professional group. However, our sample of NICU nurses had a lower depression score than published samples of emergency (28), ICU (18) or other nurses (18, 55, 58). To our knowledge, depression in midwives has not previously been measured and more research is therefore needed.

Burnout levels were similar in midwives and NICU nurses respectively: emotional exhaustion (64.7, 69%), depersonalization (37.0%, 35.7%), and low personal achievement (56.3, 60.7%). However NICU nurses were more likely to reach the severe threshold for the three subscales. The emotional exhaustion subscale is known to reflect the impact of work-related chronic stress (59) and these results are in line with previous literature on hospital midwives (10, 11) and ICU nurses (60). Our findings concerning low personal achievement in NICU nurses are in line with findings in Scottish ambulance personnel (52); regarding midwives, our scores are higher than previously found, with 10 or 30% reporting low personal achievement (10, 11). Those important percentages seem to reveal a deeper problem linked with job satisfaction likely caused by a chronically stressful working environment. Indeed, significant correlations between the three MBI subscale scores and job satisfaction in nurses have previously been published (61).

The results of this study add to the existing literature on the mental health of healthcare professionals, as research on NICU nurses in particular is scarce. The study also adds to the knowledge of the exact nature of the work-related stressors that these professional groups encounter in terms of traumatic vs. non-traumatic stressors. Another strength is the mixed-methods research design and the use of valid and reliable instruments. Our study has some limitations, notably, the lack of measurement of frequency and subjective appraisal of work-related stressors, as well as of protective factors, such as coping strategies, resilience, and social support. We had a low response rate with regards to the open-ended question within the NICU nurses sub-sample. Finally, the cross-sectional design of this study does not allow for any attributions of causality.

Future research would benefit from implementing a semi-structured interview to gain more detailed information about traumatic and non-traumatic work-related stressors. Furthermore, a prospective study with a comparison group would allow understanding the longer term impact of working in a chronically stressful working environment on the development of psychopathological symptoms. Finally, investigating protective factors, such as resilience, social support, and coping strategies would be helpful.

Our results have important implications. High prevalence rates of anxiety, and burnout symptoms found in our sample of hospital midwives adds evidence to the known dilemma they face between their primary professional value of “being with the woman” and the stressors present in the hospital environment. A stronger focus during their professional training and ongoing supervision on developing strategies that enable midwives to balance these different demands seems important. In addition, given the high prevalence of STSD symptoms in this population, the teaching of coping strategies summarized under the acronym “CORES” shown to be effective to deal with anxiety linked with maternal death, represents an interesting avenue to explore (12). Given the high prevalence of STSD and burnout symptoms in NICU nurses, strategies to change the subjective appraisal of the traumatic stressors could be taught and giving them time to recover in-between frequently occurring traumatic events needs to be ensured. For both professional groups, measures taken to increase the social support at work, such as by introducing a peer support system as well as professional mediation and other resources, are likely to protect against the development of mental health symptoms. A regular screening for psychopathological symptoms might be helpful and access to professional help should be provided if necessary. These measures might not only improve the mental health but also decrease sick leave and improve the quality of patient care.

## Ethics statement

This study was carried out in accordance with the recommendations of Swissethics, ethics committee of the Canton de Vaud, Switzerland. The protocol was approved by the ethics committee of the Canton de Vaud, Switzerland (study nr: 237/2013). All subjects gave written informed consent in accordance with the Declaration of Helsinki.

## Author contributions

AH conceived of the study and its design, coordinated the data collection, participated in the data analysis, and co-wrote the manuscript. CM, J-FT, FL, SG-N, and VB participated in the conception and coordination of the study, and commented on the manuscript. CF and LJ conducted the data collection and data analysis and co-wrote parts of the manuscript. All authors read and approved the final manuscript.

### Conflict of interest statement

The authors declare that the research was conducted in the absence of any commercial or financial relationships that could be construed as a potential conflict of interest.
