# Supplementary material for: Mental Health Symptoms and Work-Related Stressors in Hospital Midwives and NICU Nurses: A Mixed Methods Study
Source: Front Psychiatry. 2018 Aug 20;9:364. doi: 10.3389/fpsyt.2018.00364 (PMC6109791; doi:10.3389/fpsyt.2018.00364)
Supplement: Supplementary file 1 [file Table_1.docx]

**Supplementary data**

Table S1. Differences between responders and non-responders in NICU nurses regarding qualitative questions

|  |  | NICU nurses who gave at least one example of work-related stressful situations | NICU nurses who did not give any example of work-related stressful situations |
| --- | --- | --- | --- |
|  | ***p*** | Mean | Mean |
| HADS anxiety | .095 | 45.7 | 36.7 |
| HADS depression | **.049** | 46.3 | 35.8 |
| **STSS total score** | **.018** | 48.6 | 35.8 |
| **STSS intrusion** | **.011** | 49.0 | 35.2 |
| STSS avoidance | .063 | 47.9 | 37.8 |
| STSS arousal | .124 | 47.1 | 38.7 |
| **MBI emotional exhaustion** | **.023** | 51.3 | 37.3 |
| MBI depersonalization | .241 | 47.9 | 41.5 |
| MBI personal achievement^1^ | .190 | 41.1 | 48.8 |

Bold: *p* < .05

^1^ A low score indicates low personal achievement and is an indicator of burnout.
